# Supplementary material for: Are carbon emissions trading and green financial instruments synergistic? -Comprehensive quantitative research based on content analysis
Source: PLoS One. 2024 Mar 7;19(3):e0298601. doi: 10.1371/journal.pone.0298601 (PMC10919601; doi:10.1371/journal.pone.0298601)
Supplement: S1 Data — (ZIP) [file pone.0298601.s001.zip › Data/地级市Z_F工作报告.zip!/╜¡╬≈/╙Ñ╠╢╩╨/└┤╫╘╣1⁄2╓┌║┼í╛┬φ┐╦╩2╛▌═°í┐_2014.docx]

各位代表：

　 现在，我代表市人民政府，向大会作政府工作报告，请予审议，并请各位市政协委员和列席会议的同志提出意见。

　 一、2013年工作回顾

　 过去的一年，在省委、省政府和市委的正确领导下，全市上下紧紧围绕建设富裕秀美宜居和谐鹰潭的目标，全面贯彻落实省委提出的“发展升级、小康提速、绿色崛起、实干兴赣”十六字方针，按照“主攻项目、决战‘三区’，凸现特色、实现跨越”的总体要求，坚持稳中求进工作总基调，统筹做好稳增长、调结构、抓改革、优生态、惠民生等各项工作，全市经济社会保持了持续健康发展的良好势头。预计全年完成生产总值560亿元，增长11%；财政总收入92.66亿元，增长16.8%；公共财政预算收入66.13亿元，增长12.4%；规模以上工业增加值322亿元，增长13%；固定资产投资400亿元，增长22%；社会消费品零售总额137亿元，增长16%；城镇居民人均可支配收入22200元，增长12%；农民人均纯收入9940元，增长12.9%。

　 （一）产业升级提质增效。“1+6”产业快速发展，全市规模以上工业主营业务收入2750亿元，位居全省第三。铜产业完成主营业务收入2500亿元、增长16%，江铜集团跻身世界500强，鹰潭（贵溪）铜产业循环经济基地获批国家“城市矿产”示范基地；水工、节能照明、眼镜等非铜产业主营业务收入实现四年翻番。贵溪成功获批国家循环经济示范城市（县）创建地区，余江工业园列入全省重点工业园区。旅游业实现较快增长，全市接待国内游客1330万人次、增长23%，旅游总收入96.5亿元、增长28.5%；龙虎山景区接待游客650万人次、增长28%，旅游直接收入2.77亿元、增长30%。商贸物流业增势强劲，货运产业实现营业收入229亿元、增长69.4%，上缴税费9.82亿元、增长95.2%。经济运行质量更趋优化，二、三产业比重提高0.9个百分点，税收占财政收入的比重达80.3%，提高9.6个百分点。

　 （二）城市建设步伐加快。开展了城市总体规划实施评估工作，完成了信江新区南北区、高新区、老城西区等一批控规及专项规划编制。信江新区建设稳步推进，路网二期和三期、龙虎山北路、城市防洪堤、信江大市场、滨江景观一期和二期、信江浮桥等工程全面竣工，高铁站前广场、中央商务区、高教园区、市民广场等重点项目有序推进，滨江“天际线”初具雏形。老城区功能不断完善，林荫西路全面贯通，建设路、站江路、四海路和小街小巷等道路管网改造工程顺利完工，完成棚户区改造15万平方米。新建改造白鹭公园、梅园公园等十大特色公园，成功创建国家园林城市。荣膺“全国国土资源节约集约模范市”。月湖新城建设步入快车道，月湖经济大厦、绿地大道等21个项目全面推进。贵溪东城区建设步伐加快，成功创建省级园林城市。余江县城区东拓稳步推进，城市框架进一步拉大。全市建成区面积拓展到68.3平方公里，城镇化率达53%，提高1.75个百分点。

　 （三）“三农”工作稳步推进。成功应对“6·29”洪灾和历史罕见伏秋连旱，全年粮食总产达69.6万吨，实现“十连丰”。白鹤湖国家现代农业示范区创建工作进展顺利，贵溪、余江省级现代农业示范区建设初见成效，市级以上农业龙头企业158家，实现产值62.2亿元，新增农民专业合作社206家、家庭农场125个。镇村联动建设扎实推进，贵冶周边新农村建设精品示范区和“三区三线”民居改造提升工程顺利完工，全市236个“秀美乡村”建设点全面完成。实施水利建设工程项目130个，完成99座小(2)型以上水库除险加固。改造农村公路284公里。大力推进“森林城乡、绿色鹰潭”建设，完成造林绿化5.4万亩。全面启动净空、净水、净土行动，扎实推进农业面源污染治理、重金属污染防治和水库水质专项治理工作，主要河流断面Ⅱ-Ⅲ类水质和集中式饮用水源水质达标率100%，空气环境质量保持在Ⅱ级标准以上。

　 （四）开放水平明显提高。全年引进省外5000万元以上项目资金197亿元、增长14%；实际利用外资1.9亿美元、增长12.7%。招大引强步伐加快，成功引进中能石油、霸王衡器等投资5亿元以上项目12个，百炼集团、金帝鞋业等15个亿元以上项目建成投产，上诠电信、海尚科技等21个亿元以上项目开工建设。对外贸易增势喜人，连续四年被评为“全国外贸百强城市”，全年完成外贸出口9.1亿美元、增长12.3%；宁波至鹰潭转关线路正式开通，鹰潭至宁波“五定班列”运行平稳，进口固体废料6000标箱、增长151.6%；鹰潭海关、国检即将挂牌。“三争”工作成效显著，贵溪、余江纳入赣南等原中央苏区振兴规划，铜产业循环经济区、鹰潭创建本科院校等25个重大项目纳入赣东北扩大开放合作战略支持范围。

　 （五）民生保障更加有力。全市共投入民生资金18.9亿元，全面完成了省政府下达的76件实事94项民生指标。全市新增城镇就业2.39万人，城镇就业率达96.5%，新增转移农村劳动力2.03万人，发放小额担保贷款3.4亿元，我市被评为全国民营企业招聘周组织工作突出城市。连续9年提高养老金标准，人均月增资158元，发放城镇退休职工养老金7.2亿元。47.5万人参加新型农村和城镇居民社会养老保险，10万名60岁以上老人领取养老金，城乡居民参保率达95%。城镇职工医保、城镇居民医保和新农合“三张网”医疗保险体系实现城乡居民全覆盖，城镇医保水平进一步提高。发放城乡低保1.06亿元，城乡低保月人均补助水平分别达到253.7元和126.3元。新开工建设各类保障性住房4008套，基本建成2092套。累计为5292名白内障、193名唇腭裂、401名先天性心脏病、41名白血病和343名尿毒症等重大疾病患者实施免费救治。市残疾人康复中心基本建成。

　 （六）社会事业协调发展。科技创新“136”工程取得新成效，成功申报省可持续发展实验区，连续14年被评为“国家科技进步先进市”。促进教育公平取得明显进步，贫困家庭学生资助实现全覆盖，学前教育不断加强，城区学校“大班额”治理工作进展顺利。卫生服务能力进一步提升，食品药品安全监管体制改革、县级公立医院综合改革有序实施，全市100%的行政村实施了基本药物制度。文化事业日益繁荣，成功举办第二届中华赏石展暨黄蜡石文化博览会和中国第十五届根艺石艺博览会，《道之韵》赴埃及成功汇演，建市30周年系列群众文化活动丰富多彩，《龙虎山》特种邮票顺利发行。成功举办市第七届运动会和首届体育文化节，连续18年荣获全国全民健身活动优秀组织奖。深入推进平安鹰潭、法治鹰潭建设，有力打击各类违法犯罪活动，“六五”普法中期工作成效明显，社会治安、信访秩序进一步好转，安全生产形势平稳，社会保持和谐稳定。

　 （七）发展环境不断优化。政府职能转变取得明显成效，市民服务中心正式运行，251项审批服务事项集中办理，公共资源项目实现网上交易。精简行政审批项目44项，承接省下放行政审批事项66项，取消评比达标表彰项目86项。严格厉行节约，公用经费在年初预算的基础上压缩10%，“三公”经费压缩20%以上，政府投资项目优化节俭资金8亿元。集中开展企业发展环境和行业不正之风突出问题等专项整治活动，创新农村集体“三资”、民生资金和政府投资工程项目标后监管模式，审计监督、惩治和预防腐败体系建设取得新成效。

　 与此同时，人口计生、方志档案、民族宗教、外事侨务、涉台事务、气象服务、防灾减灾、质量监督、民兵预备役、国防动员、拥军优属、人民防空、妇女儿童、老龄等工作取得了新的成绩。

　 各位代表，成绩和进步来之不易。这些成绩的取得，是省委、省政府和市委正确领导的结果，是市人大依法监督、市政协民主监督的结果，是全市人民锐意进取、共同努力的结果。在此，我代表市人民政府，向全市广大干部群众，向各位人大代表、政协委员、各民主党派、工商联、无党派和社会各界人士，向驻鹰单位、人民解放军、武警官兵、公安民警和离退休老同志，向所有关心、支持、参与鹰潭经济社会发展的各界人士，表示衷心的感谢并致以崇高的敬意！

　 回顾过去一年的工作，我们清醒地看到，我市经济社会发展还存在不少困难和问题。一是经济总量不大、产业结构不优、资源环境压力加大，加快发展、转型升级仍是我们的主要任务。二是中心城区辐射带动力不强、区域经济发展不平衡、城镇化水平不高，统筹城乡协调发展仍是我们亟待加强的工作。三是教育、医疗、住房、社会保障等民生工作与群众期盼还有较大差距。四是维护社会和谐稳定、改进干部作风等方面问题仍需我们努力破解，等等。对这些困难和问题，我们将高度重视，采取有力措施，认真加以解决。

二、2014年工作安排

　 2014年是全面贯彻落实党的十八届三中全会精神、全面深化改革的第一年，改革任务重大而艰巨。做好今年的工作，对全面完成“十二五”规划至关重要。

　 今年政府工作的总体要求是：全面贯彻落实党的十八大、十八届二中、三中全会和省委十三届七次、八次全会精神，按照“主攻项目、决战‘三区’，凸现特色、实现跨越”的总体要求，坚持稳中求进、改革创新，以“8+1”重点工作为抓手，突出转型升级、城乡一体、和谐惠民、绿色发展四大工作主题，强化项目带动、开放推动、消费拉动三大发展支撑，促进经济持续健康较快发展、社会和谐稳定，奋力迈出建设富裕秀美宜居和谐鹰潭新步伐。

　 2014年全市经济社会发展主要预期目标是：生产总值620亿元，增长10%左右。财政总收入105.2亿元，增长13.5%；其中，公共财政预算收入74.7亿元，增长13%。规模以上工业增加值340亿元，增长12%以上。固定资产投资480亿元，增长20%。社会消费品零售总额159亿元，增长16%。城镇居民人均可支配收入24400元，增长10%。农民人均纯收入11000元，增长11%。居民消费价格总水平涨幅控制在3.5%左右。人口自然增长率控制在7.6‰以内。万元生产总值能耗下降3.4%，化学需氧量、二氧化硫、氨氮、氮氧化物排放量控制在省下达目标以内。

　 实现上述目标，今年重点抓好以下八个方面的工作：

　 （一）全面深化改革，增强发展活力。

　 坚持问题导向，把改革创新贯穿于经济社会发展各个领域各个环节，以改革促发展，以改革助转型，以改革增效益，以改革惠民生，激发各类市场主体和社会主体的活力，增强发展内生动力，推动经济更有效率、更加公平、更可持续发展。

　 1.加快转变政府职能。深化行政审批制度改革，巩固“两集中、两到位”改革成果，继续大力度取消和下放一批行政审批事项，优化行政审批流程，建立行政审批事项目录管理制度，完善并联审批、网上审批制度，落实重大产业项目绿色通道机制。强化行政服务中心职能，做到“一站式”审批、“一条龙”服务、“一窗口”出证。深化大部门制改革，强化机构和职责整合，理顺权责关系，严控机构编制，完成市县政府机构改革。稳妥推进事业单位分类改革。

　 2.完善现代市场体系。充分发挥市场在资源配置中的决定性作用和更好发挥政府作用，完善项目审批核准办法，鼓励支持社会资本进入基础产业、基础设施、金融以及教育、卫生、文化等社会事业领域，加快市政、园林绿化等政企分开步伐，推进市公交公司股份制改造。优化政府投资结构，重点用于具有全局性、基础性、战略性的重大项目，主要投向基础设施、高新产业、生态环保、社会事业等领域。实行统一的市场准入制度，探索负面清单管理模式，推进工商注册制度便利化，积极推行“先照后证”登记制、注册资本认缴登记制，放宽企业经营范围登记，最大限度激发民营经济发展活力。

　 3.深化财税体制改革。推进市县部门预算改革，加快建立规范统一的部门预算体系，加强预算绩效管理，增强政府统筹资金能力。深化税收征管改革，推进综合治税平台建设，加强重点行业及重点税源管控，提升征管效能。稳步推进国库集中支付电子化管理改革。有序推进“营改增”试点工作。积极推进政府向社会力量购买公共服务，制定政府向社会组织购买服务目录，建立公平开放的公共服务购买制度。切实抓好政府债务风险管控工作，探索构建政府债务预算管理体系。合理配置财政资源，优先安排社保、教育、科技、卫生等民生支出，从紧编制“三公”经费预算，扎实推进“三公”经费公开，将县（市、区）纳入公开范围。

　 4.健全城乡发展一体化体制机制。深化户籍制度改革，实行差别化落户政策，制定与户籍制度改革相适应的配套政策措施，推进以人为核心的城镇化步伐。推进农村土地承包经营权确权登记，建立健全市、县、乡三级土地流转服务体系。完善农村宅基地管理制度，确保完成宅基地使用权地籍调查。推进农村集体经营性建设用地流转改革试点工作。建立城乡统一的建设用地市场，完善土地租赁、转让、抵押二级市场。开展农村建房“三清理一整治”和闲置土地清理整治专项行动，提高土地节约集约利用水平。进一步规范征地程序，改革完善征地制度，建立兼顾国家、集体、个人的土地增值收益分配机制，落实被征地农民的社会保障。创新城市管理方式，完善城市治理结构，强化城管委职能，完成“数字城管”建设，推行城市网格化管理模式，提高城市智能化、精细化管理水平。

　 （二）致力转型升级，壮大工业实力。

　 坚持以建设绿色世界铜都为目标，以打造沿320国道产业集聚升级带为载体，以做大做强“1+6”产业为方向，着力构建“大龙头引领、大产业支撑、大集群发展”的现代工业体系，力争全市工业主营业务收入突破3100亿元。

　 1.推进产业升级。坚持“地企合作、两头延伸、转型升级”，进一步巩固和拓展铜产业在全国的领先优势，构建铜原料、铜冶炼、铜加工、铜贸易、铜研发、铜检测、铜文化为一体的产业体系，力争全市铜产业主营业务收入突破2800亿元。做精铜深加工，力争上诠电信、科炬新材料等6家企业建成投产；做强优势铜企业，力争3年内红旗集团、建鑫铜业等4家以上企业产值突破50亿元；做大铜现货交易，尽快建成铜交割库，争取设立铜期货交割仓库，形成有色金属交易市场；做旺铜原料市场，加快铜拆解加工区二期工程、江铜再生资源30万吨拆解项目建设，推进铜拆解作业区与九江港的合作，开展与海西口岸的对接，力争全年铜贸易货物量达12万吨，进口固体废料6500标箱。围绕培育壮大六大特色产业，全力打造水工、节能照明、眼镜、雕刻等十大特色产业基地，力争省级产业基地达到7个，10亿元以上龙头企业达到8个。加快发展战略性新兴产业，确保三川水表2月份整体搬迁至水工产业园，力争阳光照明、百炼化工氟化新材料等36个新上项目投产见效，河北养元年产20万吨核桃露生产基地、连展科技年产2亿支精密电子连接器等24个重大项目开工建设；力争3年内水工业、节能照明、大健康产业主营业务收入均突破100亿元。

　 2.强化平台升级。按照“特色产业带、田园风光带、宜居宜业区”的要求，精心编制规划，高标准打造沿320国道产业集聚升级带，加快形成产业发展整体优势，力争全市工业园区主营业务收入达1300亿元。推进产城融合发展，高标准实施320、206国道拓宽改造工程，加快园区道路管网、供电供水等基础设施建设，开工建设龙岗商务服务区，力争电镀集控区2月份投入运营。推进园区扩园升级，力争高新区拓园3平方公里以上，余江工业园扩区4平方公里以上，支持贵溪工业园区创建国家级工业园区。加快高新区小微企业孵化基地、童家创业基地、科技孵化器等创业平台建设，促进中小微企业快速健康发展。深入实施“园区再造工程”，盘活闲置土地和厂房，提高入园项目投资强度，力争高新区投资强度达到150万元/亩，新建标准厂房10万平方米。强化发展要素保障，搭建工业园区投融资平台和银企合作平台，发挥“财园信贷通”融资优势，设立战略性新兴产业发展专项基金，强化领导联系企业帮扶工作机制，破解企业融资、招工等发展难题。

　 3.深化创新升级。大力实施创新驱动战略，加快建设铜产业工程技术研究中心、铜新材料产业院士工作站、三川水表国家企业技术中心、国家级大健康检验检测中心等技术创新公共服务平台。大力推进省可持续发展实验区建设，实施高新技术企业倍增计划，力争新增高新技术企业15家以上，高新技术产业增加值增长20%以上。积极推进工业化、信息化深度融合，加快智慧鹰潭、电信光网城市等工程建设，提高经济社会信息化服务水平。大力实施“新市民”工程，制定企业优秀人才保障性住房、子女就学等优惠政策，营造良好的创业环境。推进知识产权“四个一”升级工程，实施质量兴市战略。

　 （三）优化产业结构，做旺现代服务业。

　 围绕打造全国区域性物流节点城市、全国著名休闲旅游目的地城市，大力发展高铁经济，培育一批拉动力强的消费增长点，推动服务业发展提速、比重提高、水平提升，力争服务业增加值占生产总值比重提高2.1个百分点。

　 1.加快推进商贸物流业大繁荣。按照“城内大商贸、城郊大市场、城乡大流通”的布局，全面推进现代物流园、鹰潭国际商贸园、铜循环经济国际物流中心三大片区建设，加快推进十大专业市场建设，力争浙商联盟建材家居城、手拉手国际汽车城、国际眼镜城、胜华机电五金商贸城、万寿宫商城等5个项目基本建成运营，开工建设物流基地、金属交易市场等一批项目。结合棚户区和城中村改造，优化城市商业网点布局，引进全国知名的大型连锁零售企业进驻鹰潭，加快建设铜锣湾等一批城市商业综合体，力争碧海蓝天、滨江广场等综合体开业运营。加强与中国网库集团合作，建设赣东北电商谷，大力发展第四方物流，力争林安物流等一批行业龙头企业早日开工。完善物流配套设施，加快铁路口岸作业区、电子口岸、保税区、物流信息平台建设。深入实施“万村千乡”市场工程，加快农村商品配送中心、乡镇商贸中心、农贸市场改造升级。

　 2.加快推进旅游业大发展。大力实施旅游强市战略，按照“一廊三区，四核多片”的规划布局，推进十大旅游项目建设，致力在丰富夏天避暑、冬天养生旅游产品上下功夫，致力在完善旅游配套、挖掘道教内涵上下功夫，努力把龙虎山打造成四季宜游的经典景区，力争全市旅游接待人次突破1500万，实现旅游收入超120亿元。全力推进在建重大旅游项目，确保龙虎山大型实景演出、逍遥城旅游综合体、圣井山综合开发、梦幻乐园等4个项目基本建成运营。完成旅游产业园区规划编制并启动基础设施建设。加快天元度假区、龙虎山大道延伸段等配套设施建设，提升旅游综合承载能力。全力办好国际道教论坛，加快大上清宫二期和道学院等一批道教宫观建设，启动龙虎山道教养生园建设，促进道教文化与旅游的深度融合。强化旅游市场营销，加强跨区域旅游协作，共同打造赣东北旅游圈、闽浙皖赣无障碍旅游黄金走廊，进一步提升鹰潭旅游业的品牌影响力和市场美誉度。

　 3.加快推进新兴服务业大提升。大力发展现代金融业，建立完善信贷政策执行和信贷投放考核激励机制，加强“信用鹰潭”建设，进一步优化金融生态环境，推进中小企业信用示范区、融资超市建设，鼓励支持优强企业上市，切实增强金融服务实体经济能力，力争引进全国性股份制银行、城市商业银行各1家，设立村镇银行1家，新开办2家以上小额贷款公司，组建政策性担保公司3家。积极推动保险业发展。加快发展文化休闲娱乐业，全力推进文化创意产业园建设，尽快繁荣特色商业街、余江雕刻一条街、黄蜡石专业市场，打响鹰潭文化创意品牌。大力发展健康养老服务、家政社区服务等新兴业态，积极发展信息咨询、企业策划等中介服务业，引导房地产业健康发展。

　 （四）突出全域鹰潭，加快城乡一体。

　 坚持以“全域鹰潭”规划为龙头，按照“1+3+10”城镇发展模式，统筹推进中心城区、县城、中心集镇、中心村“四位一体”发展，着力构建城镇功能互补、空间布局优化、产业配套协作的城乡一体化发展格局，力争城镇化率达55%。

　 1.进一步提升中心城区承载力。编制完成全域规划，完善老城区控制性详细规划，完成高铁片区修建性详规编制。信江新区重点抓好高铁北站周边、高教园区、滨江沿线三大片区建设，全面建成高铁北站站房、高铁站前广场、鹰北公路客运和公交枢纽站等6个项目，大力推进中央商务区、信江生态公园等25个项目建设，开工建设路网四期、余信贵大桥、民悦佳园等6个项目，高标准打造滨江“天际线”，聚旺新区人气。老城区按照“大片区规划、大项目推进、大配套建设”的要求，重点抓好盐业公司、二二三地质队等5个棚户区和朱埠杨家、夏家等15个城中村整体改造，搭建棚户区和城中村改造投融资平台，统筹公建项目、商贸流通、公益事业，科学布局文教卫体等公共服务设施，建设一批小广场、小游园，打造“15分钟市民城市生活圈”，达到改造一片、提升一片、繁荣一片的整体效果。鹰东片区重点实施环城东路至鹰雄大道延伸改造、中铁十一局仓储等项目。鹰南片区重点抓好天洁东路延伸、平安路改造和南站货场周边环境整治等项目。鹰西片区重点抓好鹰西高速路口周边整治、道路管网基础设施、中童镇集镇提升改造等项目。通过2-3年的努力，着力构建“新城区聚人气、老城区提品位、月湖新城和鹰西、鹰南片区竞相发展”的格局。

　 2.统筹城乡协调发展。加快发展县域经济，实施工业转型升级、现代农业“接二连三”、服务业倍增、县城建设提升、镇村联动发展、城乡环境治理“六大工程”，完善财税、土地、金融等扶持政策，进一步增强县域经济发展活力。大力推进镇村联动，重点抓好锦江、塘湾、童家、上清等十强中心镇建设，规范、用好城乡建设用地增减挂钩政策，赋予中心镇部分县级经济社会管理权限，精心规划建设核心街区、重要建筑群，大力发展特色产业集群，不断提高小城镇承载辐射能力，力争中心镇建设走在全省前列。完成200个以上自然村点的新农村建设任务，着力打造320、206国道沿线新农村建设精品通道和中心镇周边中心村精品示范区。

　 3.加快发展现代农业。加大强农惠农富农政策力度，深入开展“粮食稳产增产十大行动”，建设高标准农田3万亩，确保粮食总产稳定在68万吨以上。加快推进农业规模化，积极推行土地规模经营，加快贵溪、余江省级现代农业示范区建设，理顺白鹤湖现代农业示范区管理体制，力争成功创建国家级示范区。加快推进农业产业化，坚持市场化理念、工业化办法，培育新型农业经营主体，大力发展畜牧、蔬菜等特色农业产业，着力实施林下经济“12233”工程，扶持一批农业龙头企业，力争市级以上龙头企业新增25家以上，销售收入达70亿元以上，新增农民专业合作社200家、家庭农场100家。加快推进农业标准化，加大“三品一标”申报认证力度，新增无公害农产品、绿色食品、有机食品和地理标志农产品12个以上，建成畜禽标准化规模养殖场60个。

　 （五）强化项目带动，扩大有效投入。

　 坚持以“8+1”重点工作为抓手，深入实施项目带动战略，以大项目促进大投入，以大投入实现大发展，不断增强综合竞争力和发展后劲。

　 1.加快重大项目建设。全年计划安排重大建设项目370个，总投资840亿元，当年计划完成投资304亿元。围绕促进产业升级，重点实施工业、旅游、物流等一批支撑能力强、事关长远发展的重大产业项目159个，完成投资182亿元。围绕推进城市建设和城镇化进程，重点实施老城区道路管网改造、人防工程等一批重大城建基础设施项目68个，完成投资41亿元。围绕提升农业综合生产能力，加快实施小农水重点县、苗木花卉基地等重大农业基础项目54个，完成投资19亿元。围绕保障改善民生，加快实施保障性安居工程、市社会福利中心等一批重要民生项目34个，完成投资15亿元。围绕加快节能减排和循环经济发展，着力实施第七类废料拆解、污水处理设施等重大项目42个，完成投资32亿元。围绕完善交通、能源基础设施，着力实施白鹤湖大道、城市燃气管网等重大项目13个，完成投资15亿元。

　 2.完善项目推进机制。本着“工作项目化、项目目标化、目标责任化”的原则，实行“五个一”重点项目保障机制，对“8+1”各重点项目建立责任、协调、调度、督查、反馈、奖惩等六大机制，形成“储备项目抓谋划、签约项目抓落地、开工项目抓进度、竣工项目抓运行”的良性循环。加大土地收储力度，制定完善土地收储和供应计划，完成新增收储土地1万亩并保持每年库存储备土地1万亩，为项目建设提供充足的用地保障。本着“量力而行、尽力而为”的原则，切实加强招投标、资金、项目质量等监督管理，实现项目科学高效运作。

　 3.提高对外开放水平。抢抓实施赣东北扩大开放合作战略和央企入赣的重大机遇，以敢开放、真开放、全开放的胸怀和气魄，在更大范围、更宽领域、更高层次深化多区域开放合作交流，全面构筑大开放战略空间。围绕“1+6”产业、旅游业、商贸物流业、现代农业的做大做强，采取重点客商、重点项目“一对一”对接洽谈、跟踪落实的方式，建立招商专家咨询团、市县联合招商团、项目推进服务团，力争在引进“大央企、大省企、大外企、大民企”上取得新突破，每个产业引进1-2家龙头型企业，全年引进省外5000万元以上项目资金227亿元，实际利用外资2亿美元。实施市场多元化战略，扩大外贸规模，确保全年实现外贸出口9.78亿美元。加大“三争”工作力度，争取赣南等原中央苏区、“城市矿产”示范基地、棚户区改造等重大项目获得上级资金支持，争取规划建设通勤机场。加强项目包装储备，确保重大项目有序接替，做到谋划一批、在建一批、储备一批，力争更多重大项目列入省重点项目调度。

　 （六）保障改善民生，推进社会建设。

　 大力推进以保障和改善民生为重点的社会建设，加快完善基本公共服务体系，不断提高城乡居民生活水平，努力提升人民群众的幸福感和满意度。

　 1.突出抓好民生工程。坚持“保基本、兜底线、促公平、可持续”的原则，进一步加大民生投入，努力做到财力向民生倾斜、政策向民生聚焦、服务向民生覆盖。在全面完成省下达民生工程任务的基础上，围绕解决好人民群众最关心、最直接、最现实的利益问题，全力做好扶持创业促进更高质量就业、提高社会保障救助水平、促进教育公平和均衡发展、强化医疗卫生保障、发展公益文化、加快保障性住房建设、改善居民出行条件、实施安全惠民“菜篮子”、营造宜居生活环境、深化平安鹰潭创建等十大惠民工程64件实事，让老百姓更多更好地分享改革发展成果。

　 2.全面推进社会事业。深化教育领域综合改革，统筹城乡义务教育资源均衡配置，推进义务教育学校标准化建设和农村义务教育薄弱学校改造，巩固提升学前教育，大力发展职业教育，推动创建鹰潭本科院校。深化医药卫生体制改革，加快贵溪县级公立医院改革试点步伐，基本建成市人民医院信江新院，加快儿童医院和市人民医院全科医生临床培养基地建设，启动新农合大病保险和居民健康卡发放试点工作。健全公共文化服务体系，推进文化惠民工程，启动科技馆、新博物馆等文化设施建设，抓好文化精品创作和生产。加强食品药品监管，建立覆盖从生产加工到流通消费的全过程监管制度和体制。整合公共文化服务平台、食品药品检验实验室、农产品质量安全检验检测中心等资源，做到资源共享，协作共用。深入开展全民健身活动，积极备战省第十四届运动会。落实“单独两孩”生育政策，健全计划生育利益导向机制。进一步做好档案方志、民族宗教、外事侨务、涉台事务、气象和老龄工作，加强国防教育、人防民防、民兵预备役建设，支持各民主党派、工商联、无党派和工会、共青团、妇联等人民团体的工作。

　 3.不断创新社会治理。深化平安鹰潭建设，加强社会治安综合治理，完善立体化社会治安防控体系，创新流动人口和特殊人群服务管理，依法防范和惩治违法犯罪活动。深化法治鹰潭建设，深入开展"六五"普法宣传教育，增强市民法治意识，加大法律援助力度。深化和谐鹰潭建设，健全重大决策社会稳定风险评估机制，规范信访秩序，健全人民调解、行政调解、司法调解“三调联动”的工作体系，加大矛盾纠纷调处力度，推进社区规范化建设和网格化管理，推动社会建设和管理重心下移。深化安全生产管理体制改革，提高预防和处置突发事件的能力，坚决遏制重特大安全事故，切实保障人民群众生命财产安全。

　 （七）构建生态文明，促进绿色崛起。

　 把生态优势作为我市加快发展的最大优势，把生态建设贯穿跨越发展全过程，促进生态经济化、经济生态化，实现经济发展与生态文明相得益彰。

　 1.大力发展绿色经济。推广循环经济发展模式，大力实施工业园区循环化、生态化改造，加快推进国家“城市矿产”示范基地、余江潢溪循环经济基地建设，全力推动鹰潭高新区创建国家低碳工业园区。切实抓好节能减排，推进国家可再生能源建筑应用示范城市建设，加快淘汰落后产能，严格控制高耗能、高排放和产能过剩行业新上项目，依法关停生产工艺落后、治理不达标的污染企业。

　 2.深入推进生态建设。围绕创建国家森林城市，推进“森林城乡、绿色鹰潭”建设，加强城市园林绿化工作，加快低产低效林改造步伐，确保完成人工造林4.4万亩。加快城市生态水系治理，加速推进鹰西国家湿地公园创建工作，启动白露河周边景观和化工厂片区、鹰西大道滨江沿线提升改造工程，高标准打造“一江两岸”十里绿色长廊，努力建设“碧水绕城、绿树融城”的生态宜居城市。严格林地红线保护管理，着力深化集体林权制度、国有林场改革，建立森林资源消长考核制度，提高生态保障能力。

　 3.不断加强环境保护。健全生态环境保护责任追究制度，开展环境污染强制责任保险试点。巩固拓展贵冶周边重金属污染防治成果，深入开展环境综合整治、工业污染治理、受污染地区生态修复等工作。启动高新区、信江新区、月湖新城3个污水处理厂建设，加快实施排污管网、雨污分流、截污工程。加强大气环境监管，全面建成PM2.5等指标监测体系并运行。加强水环境保护，大力实施“一江两河”治理、农村清洁工程、农村面源污染整治等生态工程，完成贵冶节水减排改造、六国化工渣场及循环水综合治理项目，启动海利化工、麒麟化工搬迁，完成市区与余江供水管道连通应急用水工程，确保饮用水源安全，使鹰潭的天更蓝、地更绿、水更清、人与自然更加和谐。

　 （八）注重廉洁高效，加强自身建设。

　 面对新的形势和任务，我们将以转变政府职能为核心，以提高行政效能为关键，以增强政府执行力和公信力为根本，着力建设人民满意政府。

　 1.增强创新力，建设学习型政府。坚持用中国特色社会主义理论体系和党的十八届三中全会精神武装头脑、指导实践、推动工作，不断提高驾驭市场经济、应对复杂局面和推动科学发展的能力。坚持把解放思想贯穿于政府工作全过程，善于打破思维定势，敢于冲破利益藩篱，勇于突破狭隘视野，注重运用改革创新的办法解决发展中的困难和矛盾，不断增强政府工作的预见性、主动性、针对性。

　 2.增强执行力，建设服务型政府。结合推进政府机构职能转变，简政放权、缩时增效，把工作重心转到创造良好发展环境、提供优质公共服务、维护社会公平正义上来。完善市、县、乡、村四级政务服务体系，规范便民服务中心运行模式。强化敢于负责、敢于担当的意识，完善政府绩效管理，狠抓督查落实，使政府工作更加务实、亲民、高效。

　 3.增强公信力，建设法治型政府。依照法定权限和程序行使权力、履行职责，依法管理经济社会事务，规范政府行政行为。主动接受人大法律监督、工作监督和政协民主监督，自觉接受群众监督和舆论监督。坚持公众参与、专家论证和政府决策相结合，促进政府决策科学化、民主化、法制化。

　 4.增强自律力，建设廉洁型政府。深入开展党的群众路线教育实践活动，践行“八项规定”，坚决抵制“四风”，坚持勤俭办一切事业，严格遵守廉洁从政有关规定，加强行政监察和审计监督，强化对行政审批、招标投标、土地出让、政府采购等重点领域和关键环节的监管，更加科学有效地防治腐败，做到干部清正、政府清廉、政治清明。

　 各位代表，“中国梦”的伟大征程鼓舞人心，“鹰潭篇”的宏伟蓝图催人奋进。让我们高举中国特色社会主义伟大旗帜，全面贯彻落实党的十八大和十八届二中、三中全会精神，更加紧密地团结在以习近平同志为核心的党中央周围，在市委的坚强领导下，进一步解放思想，改革创新，凝聚共识，攻坚克难，努力建设富裕秀美宜居和谐鹰潭，为江西迈出“发展升级、小康提速、绿色崛起、实干兴赣”新步伐作出新的更大贡献！
